# Concurrent FDG Avid Nasopharyngeal Lesion and Generalized Lymphadenopathy on PET-CT Imaging Is Indicative of Lymphoma in Patients with HIV Infection

**DOI:** 10.1155/2012/764291

**Published:** 2012-09-06

**Authors:** Yiyan Liu

**Affiliations:** Nuclear Medicine Service, Department of Radiology, New Jersey Medical School, 150 Bergen Street, H141, Newark, NJ 07103, USA

## Abstract

Patients with HIV infection often have generalized lymphadenopathy and/or other lymphoid proliferation and are at significantly increased risk for lymphoma. This study retrospectively evaluated the diagnostic value of concurrent nasopharyngeal lesion and lymphadenopathy on positron emission tomography-computed tomography (PET-CT) with fluorine-18 fluorodeoxyglucose (FDG PET-CT) imaging. The eligible cases were from patients with HIV infection and lymphadenopathy and referred for FDG PET-CT to evaluate lymphoma or other malignancies prior to pathological investigation. FDG PET-CT images and interpretation reports were correlated with clinical information and pathological diagnoses. Among 22 eligible patients, FDG avid nasopharyngeal lesions were incidentally noted in 7 on PET-CT imaging, and all had lymphomas diagnosed with subsequent biopsies (6 diffuse large B-cell lymphomas and 1 Hodgkin's lymphoma). In the remaining 15 patients with adenopathy but no visible nasopharyngeal lesion or uptake on PET-CT imaging, 9 had biopsies and lymphomas were diagnosed in 4. The patients with FDG avid retroperitoneal or intra-abdominal lymphadenopathy had a greater possibility of lymphoma, compared to those with adenopathy localized only in the upper torso. Coexistent FDG avid nasopharyngeal lesion and generalized lymphadenoapthy on PET-CT imaging are indicative of a malignant lymphoma rather than benign lymphproliferative disease or nasopharyngeal carcinoma.

## 1. Introduction

Infection with the human immunodeficiency virus (HIV) leads to selective depletion of the helper/inducer lymphocyte subset and a subsequent acquired cellular immunodeficiency. Simultaneously, B cells may demonstrate hyperactivity and proliferation [1]. Therefore, many patients infected with HIV have persistent generalized lymphadenopathy and/or other lymphoid proliferation and are at significantly increased risk for lymphoma [2]. Without histopathological evidence, the differential diagnosis is difficult when nodes are relatively small, but imperative between benign or inflammatory lymphoid activation and malignant lymphoma. 

 Positron emission tomography-computed tomography (PET-CT) with flurorine-18 fluorodeoxyglucose (FDG) has been widely used for initial staging, restaging, and monitoring of therapeutic response in lymphoma. Some preliminary studies have also revealed a promising role of FDG PET-CT in the diagnosis and identification of HIV-associated infection and inflammation [3, 4], as well as in monitoring course of HIV infection [5, 6]. In HIV-infected patients, the uptake pattern of lymph nodes might indicate anatomical sites of viral replication, and the degree of FDG uptake is related to viral load [5]. FDG uptake by the lymph nodes was also found to be inversely related to CD4 count [6]. However, sparse studies exist about the specific role of FDG PET-CT in the diagnosis of lymphoma in HIV-infected patients with lymphadenopathy. There are no reports about observation of nasopharyngeal lesion in HIV infection on FDG PET-CT imaging. This study retrospectively evaluated the diagnostic value of incidentally noted, concurrent FDG avid nasopharyngeal lesion combined with lymphadenopathy on PET-CT imaging in HIV-infected population.

## 2. Materials and Methods 

This retrospective study was approved by the Institutional Review Board of the University of Medicine and Dentistry of New Jersey. Relevant cases were identified through a search of a computerized database of patients who underwent PET-CT imaging at the Advanced Imaging Center, University Hospital between 01/2006 and 12/2010. Medical records were retrospectively reviewed for laboratory and pathological information. 

 All eligible cases were from patients with HIV infection and lymphadenopathy and referred for FDG PET-CT to evaluate lymphoma or other malignancies prior to pathological investigation. The only inclusion criterion for the case eligibility was HIV-related lymphadenopathy as PET-CT indication. A total of 22 patients meeting above criterion were identified from the database. Although some patients had anatomic imaging studies such as CT, none of them had known neoplasm or histopathological investigation of lymph nodes prior to PET-CT. No patient had clinically suspected or imaging noted nasopharyngeal lesion or disease. The patients with HIV infection and already diagnosed with lymphoma or other malignancies prior to PET imaging were excluded from the analysis. Additional 24 patients with HIV infection and extranodal abnormalities as PET-CT indications were also excluded from the study analysis. The reason of the above exclusion was because some abnormalities might be not related to HIV infection. 

 Additional search of the database did not reveal any PET-CT case with an indication of HIV infection and undiagnosed nasopharyngeal lesion.

 In all patients, combined PET-CT was performed using a PET-CT scanner (Discovery LS, GE Healthcare) and standard techniques. Patients fasted for a minimum 6 hours before PET acquisition. After confirmation of a blood glucose level of <200 mg/dL, 555 MBq (15 mCi), sterile FDG was administered intravenously followed by a radiotracer uptake phase of approximately 60 minutes. Positron emission data sets were acquired from the base of the skull to the mid thigh, for 5 minutes at each bed position. PET images were reconstructed using the OSEM (ordered subset expectation maximization) algorithm. Low-dose CT was acquired and used for attenuation correction and was fused onto the PET images for anatomic correlation. Maximum standardized uptake values (SUV_max⁡_) of lesions were recorded.

 All scans were read by two experienced nuclear medicine physicians. For the nasopharynx, SUV 2.5 was used as a cutoff to differentiate physiologic from pathologic uptake. For the lymph nodes, quantitations of the size and SUVs were recorded in the largest and/or greatest FDG avid lesion or lesions. On analysis, the images and interpretation reports were reviewed for verification of the findings and then correlated with the patients' clinical information including the viral loads, CD4 counts, and pathologic diagnoses. 

## 3. Results 

Total 22 patients or scans met the inclusion criteria. Based on the PET-CT findings, 22 patients were divided into two groups. 

**Figure 1:**
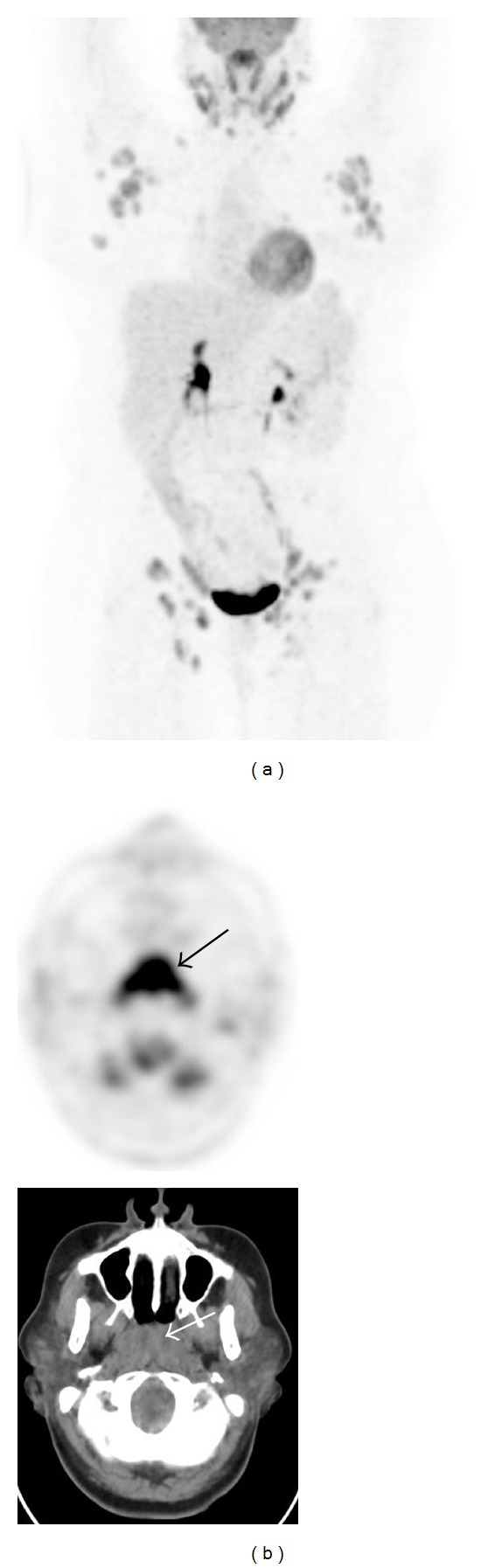
FDG PET-CT imaging of Patient 3. A maximum intensity projection image (a) shows FDG avid lymphadenopathy in multiple sites including the retroperitoneum but more prominently in the axillae and groins. Axial CT and PET images of the upper neck (b) show a nasopharyngeal mass-like lesion with intense uptake (SUV_max⁡_ 15, arrows). Subsequent nasopharyngeal biopsy confirmed DLBCL.

 Group A consisted of 7 patients with incidentally noted, concurrent FDG avid nasopharyngeal lesions in addition to generalized lymphadenopathy on PET-CT imaging.  Table 1 summarizes the patients' characteristics. The patients' mean age was 47 ± 10 years (range, 25–54 years). The range of SUV_max⁡_ in the nasopharyngeal lesions was from 5.5 to 14, and the largest lymph nodes measured from 2.0 cm to 5.0 cm. All patients underwent biopsies (5 in the nasopharynx, 1 in the neck node, and 1 in the axillary node) for histopathological diagnoses after PET-CT imaging. 6 of 7 patients had diffuse large B-cell lymphoma (DLBCL), and 1 had Hodgkin's lymphoma (HL, nodular sclerosing type). Although 2 patients had lymphoma (1 DLBCL, and 1 HL) diagnosed by biopsies of the lymph nodes in the axilla and neck, respectively, highly FDG avid nasopharyngeal mass lesions were most likely consistent with lymphoma as well.  Figure 1 is an example of FDG PET-CT images from patient 3, which demonstrate the FDG avid nasopharyngeal lesion in addition to multiple sites of lymphadenopathy.

**Figure 2:**
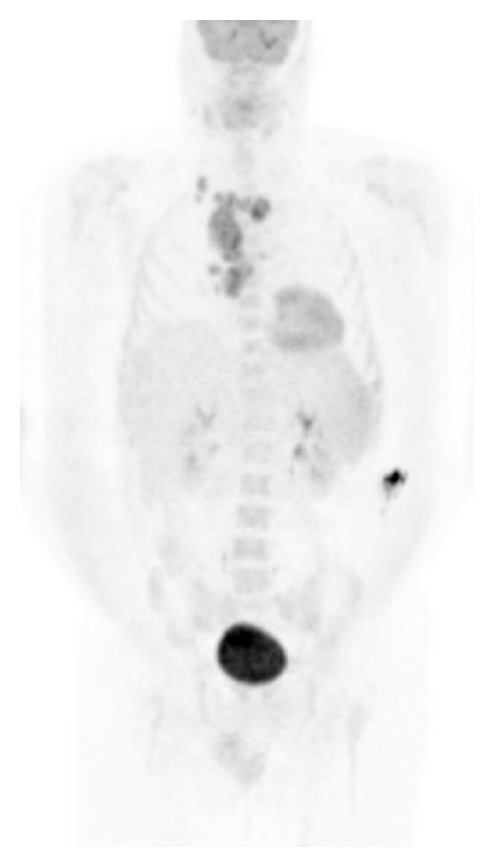
Maximum-intensity projection image of whole-body FDG PET in Patient 12. There are conglomerate mediastinal lymph nodes with intense FDG uptake (SUV_max⁡_ 12). The findings are suspicious for lymphoma. Subsequent mediastinal nodal biopsy suggested acute necrotizing inflammation.

 Group B included other 15 patients with generalized lymphadenopathy only. The patients' mean age was 47 ± 10 (range, 23–60 years). The largest node ranged from 2.5 cm to 5.1 cm (some conglomerate), and the greatest SUVs ranged from 5.4 to 12.5. In this group, no patient had visible nasopharyngeal lesion or significant FDG uptake. Some had very weak uptake, but measured SUVs were all less than 1.5. Of the 15 patients, 8 patients had lymph node biopsies and 1 had bone marrow biopsy (Table 2). Lymphomas were confirmed in 4 (3 DLBCL and 1 HL). The remaining 5 patients all had significant lymphadenopathy suspicious for lymphoma on FDG PET-CT, but biopsies were negative, which suggested that FDG PET-CT could not reliably differentiate malignant from benign lymph nodes in HIV-infected patients.  Figure 2 is an example of false positive scan in the patient 12, who had highly FDG avid bulky nodal lesions in the mediastinum, but the biopsy showed acute necrotizing inflammation rather than lymphoma.

 Additional 6 patients in Group B did not have biopsy for pathological diagnosis due to the patients' refusal or based on medical decision. In the followups ranging 2–4 years post PET-CT scans, 3 had persistent lymphadenopathy, and other 3 were lost in the followups. 

The pattern of the anatomic distribution of abnormal lymph nodes is useful for differentiating non-Hodgkin's lymphoma (NHL) from HL or benign disease, and it might be more meaningful than the size and FDG avidity of lymph nodes in HIV-infected population. As shown in Tables 1 and 2, all 9 patients with DLBCL (6 in Group A and 3 in Group B) had significant generalized lymphadenopathy in the multiple sites of both superficial (neck, axillae, and groin) and intrathoarcic/intra-abdominal locations, and involving both the upper and lower torsos. In contrast, 2 patients with HL (one in each group) and 5 lymphoma-negative cases (in Group B) all had relatively localized lymphadenopathy in the upper torso only.

Table 3 presents the mean CD4 counts and viral loads in different groups of the patients. There is no statistical difference in either CD4 counts or viral loads between the patients with and without nasopharyngeal lesions and between patients with and without lymphomas. The reason of insignificance in the statistical analysis might be secondary to the small number of the cases and very wide ranges of these laboratory results. 

## 4. Discussion

HIV infection causes depletion of CD4-positive lymphocytes with consequent immunodeficiency. HIV infection also causes, by direct or indirect mechanisms, both reactive and neoplastic changes in lymphoid tissue. Nasopharyngeal adenoidal hypertrophy is common in patients with HIV infection on pathologic study. In a report by Barzan et al. [7], 80% of 36 HIV-positive patients had pathologically confirmed nasopharyngeal lymphatic tissue hypertrophy. Most published observations suggested that nasopharyngeal lesions in HIV-infected patients were reactive and represented follicular hyperplasia [8, 9], but malignant lesions were also reported in nasopharyngeal lesions of patients with HIV infection [10]. There was a case report about malignant transformation of nasopharyngeal lymphoid hypertrophy [11]. However, to date there is no publication specifically regarding the nasopharyngeal abnormalities on FDG-PET-CT imaging in patients with HIV infection. 

 Lymphadenopathy is one of the most common and earliest presentations of HIV infection. The most common conditions affecting the lymph nodes in HIV-positive patients are reactive changes, opportunistic infections, and malignant neoplasms. Persistent generalized lymphadenopathy often precedes the development of lymphoma and is indicative of an increased risk of lymphoma [12]. The differentiation between reactive/inflammatory lymph nodes and malignant lymphoma is very challenging without histopathological investigation by invasive procedure. A few studies had suggested that FDG PET-CT is contributory to diagnosis of lymphoma and identification of both nodal and extranodal disease in patients with HIV infection. O'Doherty et al. reported that FDG-PET correctly identified 13 non-Hodgkin's lymphomas in patients with HIV infection, in which 7 with only persistent generalized lymphadenopathy, 3 with only extranodal lesions in the oropharynx, esophagus, sinus, and stomach, and 3 with concurrent nodal and extranodal lesions in the breast, joint and lung [13]. Goshen et al. also found that FDG PET-CT accurately detected lymphoma in patients with HIV infection, but 6 of 7 patients in their series had known non-Hodgkin's lymphoma prior to PET imaging [14]. In Goshen's case series, only 1 patient was for diagnosis due to lymhadenopathy, and PET finding about the lymph nodes was false positive for lymphoma. 

 Our study, for the first time, demonstrated the significance and diagnostic value of incidentally noted nasopharyngeal lesions on FDG PET-CT imaging in HIV-infected patients. In this study, 7 of 22 patients with generalized lymphadenopathy had coexistent FDG avid nasopharyngeal masses or lesions, which suggested that although nasopharyngeal lymphoid hypertrophy is commonly seen in HIV infection on pathological studies, most patients with lymphadenopathy have no visible nasopharyngeal masses or lesions or abnormal nasopharyngeal uptake on FDG PET-CT imaging. The nasopharyngeal lymphoid proliferative disease is less common than lymphadenopathy and may only appear late in the spectrum of HIV infection. However, if FDG avid nasopharyngeal lesion is present on PET-CT imaging, it is highly predictive of a malignant lymphoma. In this series, all 7 patients with concurrent FDG avid nasopharyngeal lesions and lymphadenopathy were confirmed to have lymphomas, 6 NHL, and 1 HL. In contrast, only 4 patients had lymphomas among 9 patients with FDG avid lymphadenopathy but no nasopharyngeal lesions. The findings suggested that concurrent conditions of nasopharyngeal lesion and lymphadenopathy have a higher diagnostic value for lymphoma than lymphadenopathy only and are relatively specific for lymphoma rather than a benign/inflammatory process or other neoplasms such as nasopharyngeal carcinoma. 

 After Kaposi's sarcoma, NHL is the second most common malignancy associated with HIV infection. Consistent with prior reports [15], histologies in our series were predominantly DLBCL in NHL cases, in all 9 NHL cases. Although it was reported that the most common extranodal locations of NHL in HIV infection are in the central nervous system and bone marrow [16], the present data suggested that the nasopharynx is a common site of extranodal lymphoma as well. 

 The location of lymphadenopathy may have diagnostic value for lymphoma. In the series, 9 of 9 patients with FDG avid retroperitoneal lymph nodes had DLBCL. Among other 7 patients with lymphadenopathy only located in the upper torso, 2 had HL and 5 were lymphoma-free. Extended lymphadenopathy with the retroperitoneal or intra-abdominal involvement has a greater probability for malignant lymphoma especially NHL, compared to that only localized in the upper torso such as the mediastinum, neck, and axillae.

 Our study has a limitation of small sample size. Obviously, larger studies are needed to substantiate the findings. In addition, there was a referral bias. Almost all patients had significant lymphadenopathy in the series, and those without or only mild lymphadenopathy were not referred for investigation of lymphoma with FDG PET-CT. Therefore, the incidence and significance of FDG avid nasopharyngeal lesion is unknown in patients without or with only mild lymphadenopathy.

## 5. Conclusions

Although reactive nasopharyngeal lymphoid hypertrophy is commonly seen on pathological studies in HIV infection, FDG avid nasopharyngeal lesion on PET-CT imaging may only appear late in the spectrum of HIV infection. Concurrent conditions of nasopharyngeal lesion and lymphadenopathy have a higher diagnostic value for malignant lymphoma especially NHL and are relatively specific for lymphoma rather than a benign/inflammatory process or other neoplasms such as nasopharyngeal carcinoma. 

## Figures and Tables

**Table 1:** Patients' characteristics, PET-CT findings, and histopathological diagnosis in Group A.

Patient no.	Sex/age	CD4 counts cells/*μ*L	Viral load copies/mL	PET indication	PET finding of nasopharynx	Lymph node locations on PET	Biopsy site	Pathology
1	F/52	85	848	Adenopathy	Nasopharyngeal mass, SUV 12	Neck, axillae, mediastinum, retroperitoneum, pelvis, groin; the largest 5.0 cm, SUV 18	Nasopharynx	DLBCL

2	M/51	125	1250	Adenopathy	Nasopharyngeal mass-like lesion, SUV 6.6	Neck, axillae, mediastinum, retroperitoneum; conglomerate on neck, SUV 9.9	Nasopharynx	DLBCL

3	F/48	68	2870	Adenopathy	Nasopharyngeal mass, SUV 15	Neck, axillae, mediastinum, retroperitoneum, groin; the largest 2.2 cm, SUV 12	Axillary node	DLBCL

4	F/50	62	34240	Adenopathy and otitis media	Nasopharyngeal mass, SUV 5.5	Neck, axillae, retroperitoneum, groin; the largest 2.5 cm, SUV 6.5	Nasopharynx	DLBCL

5	M/25	200	81	Adenopathy and FUO	Nasopharyngeal mass, SUV 5.8	Neck, axillae, mediastinum; the largest 2.0 cm, SUV 9.2	Neck node	HL

6	F/54	30	15900	Adenopathy	Nasopharyngeal mass, SUV 8.0	Neck, axillae, retroperitoneum, pelvis, groin; the largest 2.0 cm, SUV 6.0	Nasopharynx	DLBCL

7	M/52	Unknown	Unknown	Adenopathy	Nasopharyngeal mass, SUV 14	Neck, axillae, mediastinum, retroperitoneum, pelvis, groin; the largest 5.0 cm, SUV 15	Nasopharynx	DLBCL

SUV: standardized uptake value; FUO: fever unknown origin; DLBCL: diffuse large B-cell lymphoma; HL: Hodgkin's lymphoma.

**Table 2:** Patients' characteristics, PET-CT findings and histopathological diagnosis in Group B.

Patient no.	Sex/age	CD4 counts cells/*μ*L	Viral load copies/mL	PET indication	PET finding	Lymph node locations	Biopsy site	Pathology
8	F/50	Unknown	Unknown	Adenopathy	High FDG avid nodes	Neck, axillae, mediastinum, retroperitoneum	Axilla	DLBCL

9	F/52	234	1480	Adenopathy	High FDG avid nodes	Neck, axillae, mediastinum, retroperitoneum	Neck	DLBCL

10	M/60	191	2430	Adenopathy	High FDG avid nodes	Neck, groin, mediastinum, retroperitoneum,	Groin	DLBCL

11	F/54	70	34786	Adenopathy	High FDG avid nodes	Mediastinum, neck	Mediastinum	HL

12	M/23	26	19100	Adenopathy	High FDG avid nodes	Mediastinum, neck, groin	Mediastinum	Necrotizing inflammation

13	F/38	60	Unknown	Adenopathy, pneumonia	Moderate FDG avid nodes	Mediastinum, neck	Mediastinum	Negative for tumor

14	F/45	136	1380	Adenopathy	Moderate FDG avid nodes, esophageal uptake	Mediastinum, axillae	Axilla	Negative for tumor

15	F/42	64	Unknown	Adenopathy	Mild FDG avid nodes	Mediastinum, neck, axillae	Bone marrow	Negative for tumor

16	M/47	100	39800	Adenopathy	High FDG avid nodes	Mediastinum, neck, axillae	Neck	Negative for tumor

FDG: fluorodeoxyglucose; DLBCL: diffuse large B-cell lymphoma; HL: Hodgkin's lymphoma.

**Table 3:** The mean CD4 counts and viral load in different groups of patients.

Groups	Patients with nasopharyngeal lesions	Patients without nasopharyngeal lesions	Patients with lymphoma	Patients without lymphoma
CD4 count (cells/*μ*L)	95 ± 60	110 ± 71	118 ± 72	77 ± 42
Viral load (copies/mL)	9070 ± 13720	16496 ± 17528	10346 ± 14532	20093 ± 19229

Statistical analysis: no significant difference (*P* > 0.05) among all groups in either CD4 counts or viral loads.
